# Music and Memory in Dementia Care: Comfort With Harmony

**DOI:** 10.1093/geroni/igaa057.913

**Published:** 2020-12-16

**Authors:** Deepa Vinoo

**Affiliations:** NYC Health+Hospitals/Coler, Roosevelt Island, New York, United States

## Abstract

Data show a substantial increase in the number of people with diagnosis of dementia nationally and globally. Behavioral disturbances among persons with dementia including agitation and psychosis form a constellation of symptoms referred to as behavioral and psychological symptoms of dementia (BPSD).BPSD impacts heavily on resident’s quality of life, caregivers stress and management options for the team. FDA do not approve usage of psychotropic for Dementia related Behaviors.This study was conducted in six memory care units ,N-150.Prior to the project Psychotropic usage was 64% ,physical altercations, fallsand staff been out due to work related injury were high in those units. In 2014 a memory care team came together , identified gaps and created structured memory care units with memory care programs which includes , consistent trained staffing, meaningful engagement of residents, psychotropic stewardship program , shift from medical model to palliative model of care,person centered approach, music& memory program, shared governance, staff empowerment. All residents in those units were assessed for Psychotropic usage, falls, pain management. transfer to acute hospitals and 1;1 from 2014 to 2019. Results, Usage of Music& Memory increased from 4% 75%, Anti psychotic usage reduced by 18%, Falls reduced by 12%, Physical altercation decreased from 12 to 0, Improved pain Management .Significant reduction in transfer to acute hospitals. Reduced 1;1 from 6 to 1.Staff verbalized increased satisfaction and decreased stress. Improved family involvement. Increased bonding between residents, staff and families.Decresed work related injury while giving care.

